# Does experience provide a permissive or instructive influence on the development of direction selectivity in visual cortex?

**DOI:** 10.1186/s13064-018-0113-x

**Published:** 2018-07-12

**Authors:** Arani Roy, Ian K. Christie, Gina M. Escobar, Jason J. Osik, Marjena Popović, Neil J. Ritter, Andrea K. Stacy, Shen Wang, Jozsef Fiser, Paul Miller, Stephen D. Van Hooser

**Affiliations:** 1 Department of Biology, Brandeis University, 415 South St. MS008, Waltham, MA 02454 USA; 2 Volen Center for Complex Systems, Brandeis University, 415 South St. MS008, Waltham, MA 02454 USA; 3 Sloan-Swartz Center for Theoretical Neurobiology, Brandeis University, 415 South St. MS008, Waltham, MA 02454 USA; 4 Department of Cognitive Sciences, Central European University, Budapest, Hungary

**Keywords:** Motion, Thalamocortical, Development, Striate cortex, Area 17

## Abstract

In principle, the development of sensory receptive fields in cortex could arise from experience-independent mechanisms that have been acquired through evolution, or through an online analysis of the sensory experience of the individual animal. Here we review recent experiments that suggest that the development of direction selectivity in carnivore visual cortex requires experience, but also suggest that the experience of an individual animal cannot greatly influence the parameters of the direction tuning that emerges, including direction angle preference and speed tuning. The direction angle preference that a neuron will acquire can be predicted from small initial biases that are present in the naïve cortex prior to the onset of visual experience. Further, experience with stimuli that move at slow or fast speeds does not alter the speed tuning properties of direction-selective neurons, suggesting that speed tuning preferences are built in. Finally, unpatterned optogenetic activation of the cortex over a period of a few hours is sufficient to produce the rapid emergence of direction selectivity in the naïve ferret cortex, suggesting that information about the direction angle preference that cells will acquire must already be present in the cortical circuit prior to experience. These results are consistent with the idea that experience has a permissive influence on the development of direction selectivity.

## Background

It is apparent that many features of our behavior, and thus features of our brains, are learned. Children raised in an environment where a language such as French is not spoken do not spontaneously speak French, while children raised in a French-speaking environment typically learn to speak French. We ski and use tools that must be learned. We learn to avoid tastes of food that previously made us sick [1]. We can learn to read with substantial training [2]. Clearly, experience has a major influence on our brain.

But in this work, we consider the more specific and reduced problem of the development of basic sensory neuron receptive fields in the visual cortex of mammals. Presumably, these receptive fields are designed in order to optimally interpret the world as experienced by the array of photoreceptors on the retina, which lies at the end of an image-forming lens. That is, in the case of the visual system, these sensory neurons would examine images and extract features that are necessary for basic navigation within the environment, object segregation, predator avoidance, mate identification, etc.

One might imagine two extreme hypotheses about the development of sensory detector neurons in cortical sensory areas. In principle, there is no reason why the properties of these basic sensory detectors could not have been determined over generations of evolution and natural selection, without the need to rely on the sensory experience of an individual animal [3]. The types of images that would be observed within a given ecological niche have likely not changed considerably in hundreds of millions of years.

On the other hand, developing mammals undergo a long, protected period of parental rearing, which would enable an individual animal to have considerable sensory experience before attaining independence [4]. Therefore, perhaps the visual system can develop its sensory response properties solely using the statistics of stimuli that are experienced by an individual animal. Several unsupervised computational learning models of development have shown that neurons in randomly seeded networks, when trained with images of natural scenes, acquire receptive field properties that greatly resemble receptive field properties of neurons in primary visual cortex; they exhibit features like orientation selectivity and spatial frequency tuning [5–7]. Until the last decade, one could have reasonably argued whether the entire visual system, including cells selective for high-level features like faces, could really be built from scratch, but modern deep learning systems that are trained on enormous sets of data, such as a huge repository of images and videos on the internet, exhibit the ability to identify objects or faces (e.g., [8, 9]). These deep learning networks show similarity to the hierarchical organization of the visual system in primates, exhibiting progressively more complicated and selective receptive fields as one moves away from the analog of the photoreceptors. In the first layers, deep learning unit response properties resemble those of early visual cortical neurons, while higher layers can exhibit selectivity for classes of objects or faces, much like neurons in the inferior temporal (IT) cortex in primates [10–12].

This argument shows that a “thought analysis” of the information processing problem to be solved by the visual system (Marr’s “first level”) [13], in this case, does not usefully constrain broad ideas about nature vs. nurture with respect to how primary sensory neurons actually develop in mammals. While these thought analyses are useful in dissecting many problems in systems neuroscience, they do not offer much in the way of constraints for understanding this development. In principle, the properties of primary cortical sensory neurons could be built-in and not require the sensory experience of the individual animal, or these properties could be learned via the sensory experience of individual animals.

**Fig. 1:**
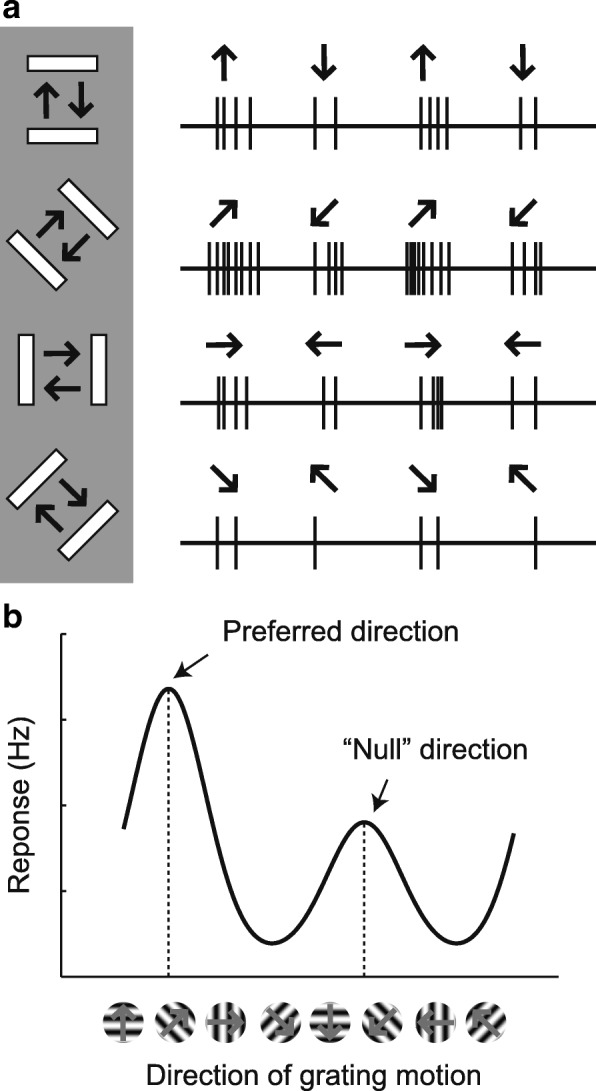
Orientation and direction selectivity in visual cortex. **a** Left: A bar visual stimulus that is swept back and forth across the receptive field of a cortical neuron. The orientation of the bar is varied over 4 angles, and the direction of motion of the bar is varied over 8 directions. Right: Responses to stimulation at different orientations and directions. This particular neuron responds to many orientations and directions, but provides particularly strong responses for stimuli moving up and to the right. **b** A tuning curve graph of the responses of the same cell as a function of direction angle. The preferred direction and the “null” direction (direction opposite the preferred) are indicated. Adapted from [36, 78]

In this review, we consider the development of receptive field properties in the primary visual cortex of carnivores. We focus in particular on the development of orientation selectivity and direction selectivity (Fig. 1), as many review articles already focus on the development of visual acuity and ocular dominance columns and the impact of deprivation such as in the disease amblyopia (e.g., [14, 15]). We present evidence from a series of recent studies from our group that suggests that while experience is necessary for the development of direction selectivity, the parameters of the direction tuning that develops are largely determined before the onset of visual experience. Therefore, we argue that visual experience has a largely permissive role in the development of direction selectivity.

## Main text

### Orientation selectivity is present at the onset of visual experience but direction selectivity requires visual experience

At the time of eye opening, neurons in primary visual cortex of cats and ferrets already exhibit substantial selectivity for stimulus orientation [16–20]. These species open their eyes postnatally, after several days or weeks of life (approximately 1 week for kittens, and 1 month for ferrets), and one may reasonably ask whether the low resolution, low contrast vision that is permitted through the closed lids, and drives visual responses [21, 22], is necessary for the development of orientation selectivity. However, orientation selectivity has also been found in ferrets that have been dark-reared from before the time that visual responses can be driven through the closed eyes [23], suggesting that no visual experience of any type is required for the development of orientation selectivity. Orientation selectivity does increase in magnitude with visual experience [16], and can be degraded by impoverished experience such as binocular lid suture [24], but all current evidence suggests that the naïve visual cortex exhibits orientation selectivity.

**Fig. 2:**
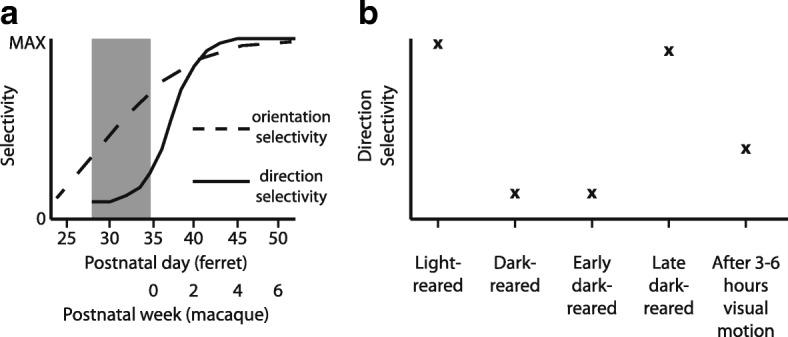
In ferrets and primates, direction selectivity develops postnatally. In ferrets, it has been shown that the development of direction selectivity requires visual experience. **a** Profile of development of orientation selectivity and direction selectivity in ferrets [19] and rough equivalent in macaque from [29]. **b** The influence of experience on the development of direction selectivity in ferret. Light-reared animals (that is, typically-reared animals) exhibit strong direction selectivity for animals P63 or older [19]. By contrast, dark-reared animals P63 or older exhibit poor selectivity for direction selectivity. Animals that were dark-reared until P45–50 and then reared under typical conditions (“Early dark-reared”) also failed to develop direction selectivity, indicating that early visual experience is required for the proper development of direction selectivity [19]. Animals that were dark-reared only until P35, and then allowed 2–3 weeks of visual experience, exhibited strong direction selectivity [19]. Finally, artificial experience with moving stimuli for 3–6 h is sufficient to cause a rapid increase in direction selectivity in visually naïve ferrets [35]. Adapted from [19]

In carnivore visual cortex, a majority of neurons are not only selective for stimulus orientation, but also respond more vigorously when a preferred stimulus moves in a particular preferred direction [25–27]. Direction selectivity is not present at the time of eye opening, and matures greatly over the subsequent 2 weeks [19] (see Fig. 2). Direction selectivity does require visual experience, as animals that are dark-reared do not acquire direction selectivity [19]. Further, there is a critical period for the development of this selectivity: animals that are dark-reared for about 2 weeks after the time of natural eye opening, and subsequently reared in typical light/dark conditions for 2–3 additional weeks, did not develop direction selectivity [19]. These typical increases in direction selectivity over the first 2 weeks after the onset of visual experience allow for a more robust discrimination of direction by downstream neurons [28].

While there has been less research on the development of direction selectivity in primary visual cortex of primates, the available evidence shows that direction selectivity also develops postnatally in macaques. The eyes of macaque infants are open at birth; orientation selectivity is already present at birth, but direction selectivity develops to adult-like levels over about the first 4 weeks [29] (see Fig. 2). Direction selectivity is organized differently in the primate, as it is largely confined to specific layers of primary visual cortex [30–32], whereas in carnivores like the cat and the ferret, direction selectivity is found to some degree in all cells in columns that span all layers. In carnivores, it appears that direction selectivity is *the* fundamental property that is mapped across the cortical surface [25, 27, 33]. While we are not aware of a physiological experiment that examines whether visual experience is necessary for the development of direction selectivity in primates (such as a dark-rearing experiment with measurements of direction selectivity), psychophysical studies in humans have shown that individuals who have experienced poor vision in both eyes have much higher motion detection thresholds than those who have had good vision in a single eye or both eyes [34], suggesting that experience may be necessary for direction selectivity in primates as it is in carnivores.

In the ferret, very little experience is required for the emergence of direction selectivity. A study that used 2-photon imaging to monitor receptive fields over time found that just 3–6 h of visual experience with a stimulus that moved back and forth was sufficient to cause the rapid emergence of direction selectivity in anesthetized ferrets [35] (Fig. 2b). These results indicated that direction-selective neurons are initially orientation-selective only, and then acquire direction selectivity with experience. By contrast, experience with a flashing oriented stimulus did not cause an increase in direction selectivity, indicating that visual experience with a moving stimulus was important for this rapid development.

Given that experience is necessary for the emergence of direction selectivity, it is interesting to ask how the parameters of direction tuning – magnitude of selectivity, direction angle preference, and speed tuning – are derived. Are these tuning parameters already specified in the naïve circuit, such that visual experience simply serves a *permissive* role to complete processes that are fully seeded at the onset of visual experience? Or, is the cortex performing some sort of information analysis on the images that it sees, and deriving these tuning parameters based on the qualities of visual experience, such that experience *instructs* the determination of these tuning parameters?

### Initial biases predict eventual direction preferences

**Fig. 3:**
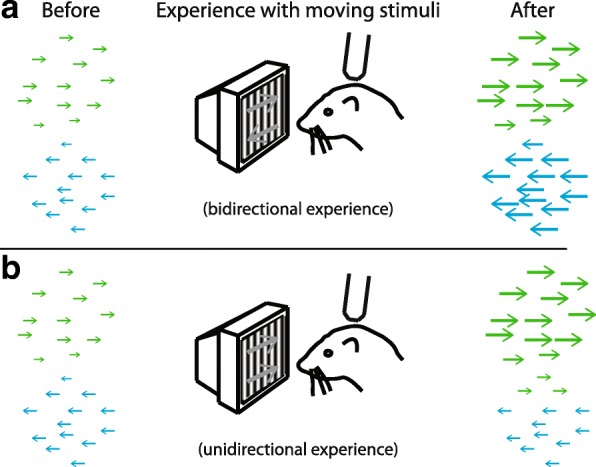
Initial biases in the naïve cortex correlate with the direction angle preference that is acquired. **a** Left: Sketch of imaging field in ferret visual cortex at the onset of visual experience. Neurons exhibit very weak direction selectivity, as indicated by small arrows. Nevertheless, there are regions that have statistically significant biases for particular directions, such as right (green) and left (blue) as shown [35]. These biases are found even in animals that have been dark-reared [23], suggesting that they are formed independent of any visual experience, including that through the closed lids. Middle: Artificial experience of 3–6 h with moving visual stimuli is sufficient to produce the rapid emergence of direction selectivity in visual cortex. In this case, stimuli moved in one of two opposite directions (random alternation), 5 s on, 5 s off, in 20 min blocks, with a 10 min rest period. Right: Sketch of imaging field after bidirectional experience, with enhancement of direction selectivity in both regions [35]. **b** Left: Sketch of initial imaging field at the onset of visual experience. Middle: Animal is provided with 3–6 h of artificial experience with moving stimuli, but here the stimuli move only in a single direction. Right: Sketch of imaging field after unidirectional experience. Neurons in regions that were biased toward the “trained” direction exhibit robust increases in direction selectivity. Neurons in regions that were biased to the opposite direction showed little change. Neurons in intermediate regions could be recruited to exhibit selectivity for the trained direction [23]

The fact that experience with a bidirectional motion stimulus could cause the rapid emergence of direction selectivity raised an interesting conundrum. There was no information in the stimulus that would have instructed the neurons to choose one particular direction to prefer over another. So, what factors caused the neurons to express a direction preference? Even though individual cells initially exhibited very weak direction selectivity that, in most cases, was not statistically significant (see [36]), an analysis of the similarity of physically nearby cells (< 100 μm) indicated that there were very slight but significant initial biases in the emerging map [35] (Fig. 3a). That is, cells in small regions of the map were more likely to exhibit similar tiny biases towards a particular direction than was expected by chance. These initial biases were predictive of which direction angle preference neurons would acquire during stimulation with a bidirectional stimulus. These initial biases were also found in dark-reared animals, suggesting that experience through the closed lids is not necessary for their formation. This is the first indication that information about some of the parameters of the direction selectivity that will emerge – in this case, direction angle preference – is already weakly present in the circuit before the onset of visual experience.

Are these initial biases immutable, in that they completely determine the direction preference that will emerge? Or can appropriate experience alter the direction preference that a cell will acquire? To address this question, we provided animals with experience with stimuli that moved in a single direction [23, 35]. We found that the direction selectivity that emerged depended greatly on the position of cells within the emerging map (Fig. 3b). Cells in regions of the emerging map that matched the direction of visual stimulation that was provided exhibited very strong increases in direction selectivity. Cells in regions of the emerging map that exhibited biases for the direction opposite to that provided exhibited no significant change in direction selectivity. And cells in regions that did not exhibit a coherent bias became slightly more selective to the direction of the visual stimulus that was provided.

This evidence is consistent with both permissive and instructive processes. The fact that neurons in regions that exhibited biases to the direction opposite to that provided in the training stimulus did not show increased selectivity indicates that these neurons did not flip their direction angle preference to match the training stimulus. This result is consistent with a permissive role for experience; those regions did not receive experience with a stimulus that matched its biases, so selectivity did not increase. However, this experiment does not settle the issue, as one could object that we simply did not provide stimulation of sufficient duration (only 3–6 h of stimulation was used) to alter these preferences. Further, neurons in regions that did not exhibit coherent biases did gain some selectivity to the trained stimulus, indicating that there may be some instructive processes that contribute to the emergence of direction selectivity.

### In principle, instructive mechanisms could be at play

In the face of inconclusive evidence for permissive and instructive processes, we sought to build a computational model that was instructive but that could also account for the reinforcement of initial biases [37]. We hoped that this model would yield experimental predictions that would provide strong tests for instructive processes.

**Fig. 4:**
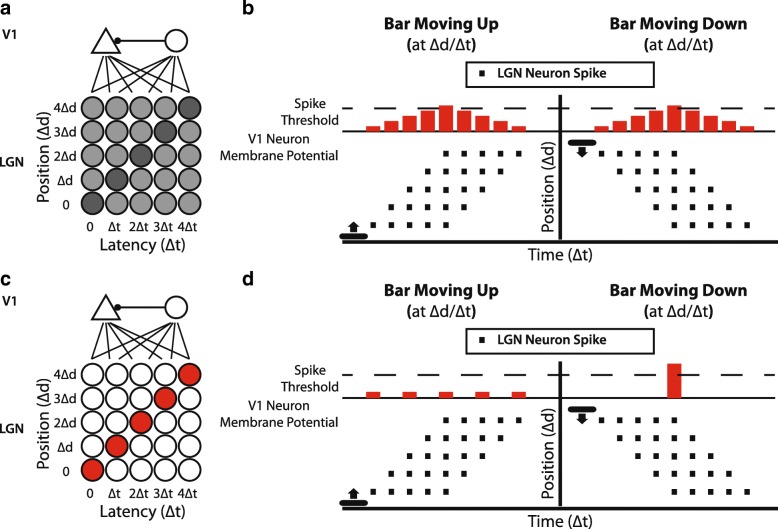
A feed-forward model with Hebbian plasticity and increasing feed-forward inhibition can, in principle, develop direction selectivity in an instructive manner. **a** Schematic of the feed-forward model [37]. An array of LGN neurons with a broad array of different position preferences and response latencies provide input to a cortical excitatory neuron and a feed-forward cortical inhibitory neuron. The cortical inhibitory neuron provides input to the excitatory neuron. The picture shows an immature network that has a slight (subthreshold) bias for downward motion (darker LGN cells indicates slightly stronger weight). Connections from LGN to the cortical excitatory neuron undergo spike-timing-dependent plasticity, while the synapse from the cortical inhibitory neuron onto the cortical excitatory neuron increases with each bout of stimulation, forcing competition among the inputs [38]. **b** Responses from the naïve model cortical neuron and LGN neurons to upward and downward stimuli. Each row of LGN cells responds to stimuli at a particular position, with varying latencies. Each black square represents the spiking activity of a single LGN cell. In the middle of the stimulus, the 5 LGN cells along each diagonal are activated simultaneously, allowing the cortical excitatory neuron to fire. **c** Connections after hundreds of bidirectional stimulation events. The increasing inhibition has forced the cortical excitatory neuron to develop selectivity for downward motion; the LGN inputs that support upward motion (the direction opposite the initial bias) are eventually weakened, because they do not drive the cell after the feed-forward inhibition has developed to full strength. **d** After training, the cortical neuron responds to stimulation in the downward direction exclusively. Stimulation with downward motion at the appropriate speed (∆d/∆t) will cause the 5 cells that comprise the diagonal to be activated simultaneously, providing strong drive to the cortical neuron. Stimulation with upward motion activates the same 5 LGN cells, but asynchronously, such that they do not drive the cortical cell. Adapted from [37, 39]

We constructed a feed-forward model of lateral geniculate nucleus (LGN) inputs onto a single V1 neuron. We represented the LGN as an array of spot detector neurons with a range of position and latency preferences, much as cells in the real LGN (Fig. 4a, b). We further postulated that Hebbian plasticity mechanisms were operating at LGN-to-V1 synapses, so that connections from LGN neurons that fired before the V1 cell would be strengthened, while connections from LGN neurons that fired after the V1 cell would be weakened. As is typical in Hebbian models, we constrained synaptic weights to be smaller than a ceiling value, to prevent runaway excitation.

We imagined that, initially, the V1 neuron would receive weak inputs from many LGN neurons with a wide range of position and latency preferences. If the neuron were provided experience with unidirectional stimulation at a particular speed, then connections from LGN neurons at the appropriate positions and latency preferences would be strengthened while other connections would be weakened, and the neuron would exhibit strong responses to the experienced stimulus. However, in the presence of bidirectional visual stimulation, the V1 neuron would become more responsive to stimulation in both of the stimulated directions, which would have resulted in a more responsive neuron but a neuron that exhibited no increase in direction selectivity, opposite to what is observed in V1 neurons in ferret visual cortex.

To address this issue, we added feed-forward inhibition to the cortex. Further, we added activity-dependent increases in the strength of the input from the feed-forward cortical interneuron onto the V1 excitatory neuron, as was observed in slice recordings in rat visual cortex [38]. This feature forces a competition among the inputs to the V1 neuron; with each stimulus, the feed-forward inhibition rises, so only those inputs that can drive the V1 neuron above the rising inhibition will persist; others will be weakened by the Hebbian plasticity. When this feed-forward inhibition was added, then bidirectional training produced a direction angle selectivity that matched the cell’s initial bias, whether that bias was subthreshold or suprathreshold (Fig. 4c, d). In addition, unidirectional stimulation still caused the emergence of a direction angle selectivity to the experienced stimulus.

This model posited several testable hypotheses: a) that the initial feed-forward LGN input to cortical neurons is diffuse and broad, with inputs from cells with a variety of spatial preferences and latencies possible; b) that Hebbian synaptic plasticity rules are operating at LGN-to-V1 synapses; c) that intracortical inhibition provides for competition among feed-forward inputs to force selectivity; and d) that, in addition to the formation of direction selectivity, the emergence of speed tuning should be experience-dependent (follows from point a).

### Short-term experience with stimuli at particular speeds does not alter speed tuning

**Fig. 5:**
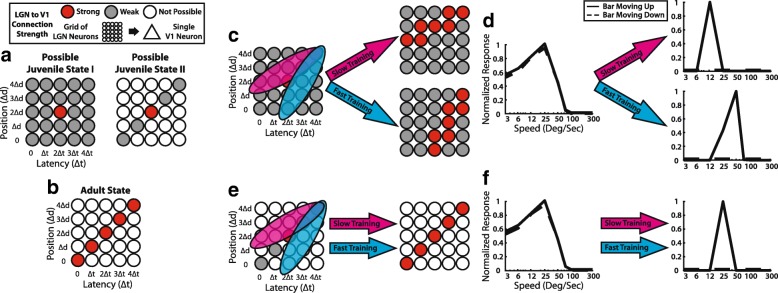
Hypotheses about initial circuit and the effect of speed training. **a** Hypotheses about initial circuit. In Possible Juvenile State I, the cortical neuron can receive input from an array of LGN cells with a wide variety of position preferences and latencies. In Possible Juvenile State II, the cortical neuron is pre-constrained to receive inputs from cells with particular position and latency values. **b** Hypothesized adult state. Neurons with particular position and latency preferences converge on the cortical neuron, resulting in direction selectivity. **c** Impact of providing experience with stimuli moving at different speeds. In Juvenile State I, only LGN neurons with positions and delays that were activated by a particular speed are strengthened, resulting in speed selectivity that matches the experienced speed. **d** Speed selectivity before and after training under Juvenile State I. **e** In Juvenile State II, the eventual speed tuning is built-in before the onset of experience, and visual experience with moving gratings merely enhances this pre-constrained tuning. **f** Speed selectivity before and after training under Juvenile State II. Experiments in ferrets strongly resembled the outcomes in (**e**) and (**f**) [39]. Adapted from [39]

In our next project, we tested some predictions of our modeling work in an experiment. To test the model feature that initial feed-forward LGN inputs were diffuse and broad – that is, that inputs from LGN cells with many possible positions and latencies were capable of providing input to individual V1 neurons – we examined whether the speed of the experienced stimulus would influence both the direction and speed tuning that would be acquired. The diffuse and broad hypothesis is contrasted with an alternative hypothesis – that inputs are initially sparse and constrained – in Fig. 5a. Under the broad hypothesis, experience with stimuli at a particular speed should cause inputs from LGN cells with positions and latencies that support responses to that speed to increase, and the neuron should acquire direction selectivity and also speed tuning that matches the experienced stimulus (Fig. 5c, d). On the other hand, if connections are initially sparse and constrained to grow according to a predetermined pattern, then the speed of the experienced stimulus should increase direction selectivity but should not influence the speed tuning that emerges (Fig. 5e, f).

To accomplish this, we measured speed tuning and direction selectivity before and after visual training in 3 groups of naïve animals that were provided with different types of visual experience. One group of animals was provided experience with moving visual stimuli that drifted at 12.5°/sec; a second group of animals was provided experience with moving visual stimuli that drifted at 50°/sec; and a third group of animals simply observed a gray screen and served as a control. Experience with either slow or fast stimuli (but not a gray screen) caused an increase in direction selectivity as expected, but the visual stimulus that was experienced by the animal did not influence the speed tuning of V1 neurons. All neurons maintained a preference for 25°/sec. This evidence suggests that information about speed tuning is already present in the visual system before the onset of visual experience, and indicates that short-term exposure to stimuli at different speeds does not alter speed tuning in V1 neurons [39]. This result suggests that cortex cannot selectively amplify inputs with arbitrary spatial position preferences and arbitrary latencies/delays, and is more consistent with the idea that the positions and latencies/delays are already specified prior to the onset of experience. That is, these data are most consistent with the picture presented in (Fig. 5e, f).

### Direct cortical stimulation causes the rapid emergence of direction selectivity

We also sought a more direct test to examine the ability of neural activity to shape – or not to shape – the parameters of selectivity of cortical neurons. The light-activated channel channelrhodopsin2 (ChR2) allows a neuron to be driven directly with light. We combined viral expression of ChR2 in the cortex of naïve ferrets with the use of a custom-built ProjectorScope that allows the image of an LCD projector to be minified and projected directly onto the surface of the cortex [40]. Because there is a retinotopic map of the visual field on the cortical surface, we could mimic a moving visual stimulus by producing a sweep of activity across the cortex.

**Fig. 6:**
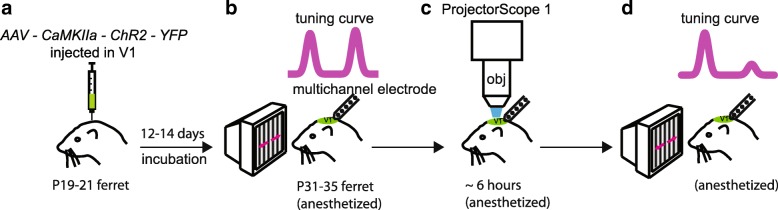
Direct cortical activation in visually naïve ferrets produced an increase in direction selectivity. **a** Engineered viruses that cause the expression of Channelrhodopsin-2 were injected in young ferrets several days before eye opening. **b** Initial orientation and direction selectivity were assessed with visual stimuli. **c** Next, the visual display was switched off and patterned light was shone on the cortex to produce specific activity patterns for several hours. **d** Periodically, the visual display was switched on and orientation and direction selectivity were assessed with visual stimulation. We found that 9 h of this direct cortical stimulation protocol resulted in an increase in cortical direction selectivity [40]. Adapted from [40]

We specifically wanted to test if neural activity that mimicked a moving visual stimulus was required to develop direction selectivity, and to further test if we could impose a direction angle preference by repeatedly providing sweeps of activity that corresponded to motion in a particular direction in retinotopic space. We initially measured orientation and direction tuning as in our previous experiments (Fig. 6a, b). Then, we provided experience with an optogenetic stimulus (Fig. 6c). In some experiments, we provided a full-field flashing stimulus (1 s on, 10s off) to see if direct cortical activation was sufficient to produce direction selectivity [40]. In other experiments, we provided a grating stimulus that drifted in a particular direction across the cortical surface. In a final group, we provided the drifting grating stimulus in animals that did not undergo virus injections for ChR2, to serve as a control.

We found that activation with either full-field flashing stimuli or drifting grating stimuli was sufficient to cause the rapid emergence of direction selectivity (Fig. 6d). Importantly, animals that did not express ChR2 did not show an increase in direction selectivity. These results imply that information about the direction preference that each neuron should acquire was already present in the circuit at the onset of stimulation, and that non-specific activation was sufficient to cause the formation of direction selectivity. Furthermore, in animals that were provided drifting grating stimulation, we found no tendency for neurons to acquire a direction angle preference that matched the mimicked visual direction of motion, suggesting that direct activity in the cortex could not, under these conditions, impose a direction angle preference [40]. This evidence is again consistent with the idea of a permissive role for visual experience in the development of direction selectivity: information about parameters such as direction preference appears to be sufficiently encoded in the initial circuit and difficult to modify.

These direct stimulation experiments raise the question of how cortical mechanisms alone, without stimulation of the retina and LGN, might underlie the development of direction selectivity. In a recent modeling study [41], we explored many models in which cortical intrinsic connections could be modified by activity in order to increase direction selectivity. In one plausible model, we imagined that local cortico-cortical connections were initially so strong that they caused the cortical response to be unselective; that is, they “blurred” feed-forward input that was selective. Cortico-cortical connections were endowed with Hebbian plasticity with a slight bias towards reducing synaptic weights on average. Activity patterns that simulated visual experience served to reduce the coupling across direction columns so that the pre-existing selective feed-forward input could be more faithfully amplified. This model reproduced the set of changes in selectivity with unidirectional and bidirectional training that was observed in vivo.

While our ProjectorScope experiments suggest that modification of cortical circuits alone is sufficient to produce increases in direction selectivity [40], it may be the case that both feed-forward and cortical-intrinsic mechanisms are at work in typical development of the visual system.

### Can the mouse help us uncover the origins of the circuit mechanisms of cortical direction selectivity?

The short answer is that it’s complicated – some aspects of the development of direction selectivity in the mouse don’t depend on visual experience. The overall development of direction-selective cells in mouse visual cortex does not depend on visual experience [42], as it does in carnivores and apparently primates. This is likely due to the fact that the mouse has a high percentage of direction-selective ganglion cells already in the retina (~ 25%), and these cells project to LGN relay cells that project to cortex [43]. Carnivore retina exhibits a much smaller percentage of direction-selective retinal ganglion cells (cats: ~ 1%) [44]. Therefore, while it is likely that any feed-forward direction-selective input channel from the retina is very small in carnivores and likely doesn’t contribute meaningfully to cortical direction selectivity, this is not the case in mice. However, it still may be possible to study direction selectivity in the mouse that arises from retina-independent channels. A recent study found that some direction selectivity remained in the cortex after partial or total genetic ablation of direction-selective retinal ganglion cells in the mouse retina [45]. Retina-independent direction selectivity could, in principle, be studied in these modified mice.

## Conclusions

We have argued that the bulk of the present evidence suggests that experience has a permissive role in the development of direction selectivity in ferret visual cortex. Experience is required in order for direction selectivity to emerge, as it does not emerge in dark-reared animals [19]. But, the experience of an individual animal apparently has a limited ability to alter the parameters of direction tuning, such as direction angle preference and speed tuning.

These recent findings immediately beg two major questions about the development of receptive field properties in the cortex.

If the parameters of direction tuning – magnitude of selectivity, direction angle preference, and speed tuning – are specified independent of visual experience, then how are these parameters specified mechanistically? Are the patterns of spontaneous activity in the developing retina, LGN, and cortex critical to establishing these parameters [46–52]? Or are these parameters determined by molecular cues that direct the early patterning of connections among these areas [53], as is the case for the initial retinotopic organization of these connections [54]?

A second major mystery that remains is to understand the circuit mechanisms that underlie the parameters of direction tuning in adult animals. In the domain of orientation selectivity, we are much more knowledgeable. The neural mechanisms underlying orientation selectivity has been reviewed elsewhere (e.g., [55]), but, for example, experimental work has identified several key features that determine orientation selectivity, including the projections of collinear LGN receptive fields onto single neurons [56–58], and push/pull of excitation and inhibition [59–62] that would be needed to allow orientation selectivity across a range of contrasts [63, 64]. Local non-linearities arising from the clustering of orientation-selective inputs on dendrites of cortical neurons may also play an important role [65]. However, we know only a little about the arrangement of LGN inputs with respect to position and latency. We know more about how the timing of inputs from excitatory and inhibitory neurons influences direction selectivity [66], but the circuit origins of these inputs are still unclear. The field has a number of hypotheses about the circuit mechanisms [37, 67–71], but the exact circuit mechanisms that underlie the emergence of direction selectivity specifically in the visual cortex of carnivores and primates remain to be determined. Knowledge of the circuit mechanisms of direction selectivity could shed light on the processes of development, just as understanding the processes of development can shed light on the circuit mechanisms in the mature animal.

It is worth noting that while we focused on basic canonical features of cells early in the visual pathway such as orientation and direction selectivity in response to full-field stimulation, neurons in the primary visual cortex are known to respond in a more intricate manner depending on the visual context of the stimulus falling directly on their receptive field [72, 73]. The traditional measures of direction and orientation selectivity do not necessarily tap into these aspects of coding. Therefore, we do not have the full picture of how the development of these more complex characteristics of selectivity might depend on early experience. Indeed, preliminary data using more sophisticated measures of developmental changes of neural behavior suggest that, while basic characteristics are left largely unaffected, experience strongly influence the emerging complex properties of cells related to integrating orientation and direction information into an internal representation [74, 75].

Interestingly, we also found recently that cross-orientation suppression and surround suppression emerged in the ferret primary visual cortex regardless of whether the animal had any visual experience [76]. Thus, a picture is emerging, in which evolutionarily determined and experience-permitted processes scaffold basic mechanisms of the visual system, such as sensitivity to orientation and direction information. We know that experience must have significant influence on the brains of individuals, as we can learn to read or learn other practiced skills. But perhaps the properties that are tuned by early experience are only observable by more sensitive measures such as natural scene responses or higher order statistics [77].

## Abbreviations

LCI: Local coherence index
LGN: Lateral geniculate nucleus
V1: Primary visual cortex
